# Validity of gestational age estimates by last menstrual period and neonatal examination compared to ultrasound in Vietnam

**DOI:** 10.1186/s12884-016-1192-5

**Published:** 2017-01-11

**Authors:** Nicholas P. Deputy, Phuong H. Nguyen, Hoa Pham, Son Nguyen, Lynnette Neufeld, Reynaldo Martorell, Usha Ramakrishnan

**Affiliations:** 1 Nutrition and Health Sciences Program, Laney Graduate School, Emory University, Atlanta, GA USA; 2 Thai Nguyen University of Pharmacy and Medicine, Thai Nguyen, Vietnam; 3 International Food Policy Research Institute, Washington DC, USA; 4 Global Alliance for Improved Nutrition, Geneva, Switzerland; 5 Hubert Department of Global Health, Emory University, Atlanta GA, USA

**Keywords:** Gestational age, Last menstrual period, Ultrasound, Neonatal examination, Vietnam

## Abstract

**Background:**

Accurate estimation of gestational age is important for both clinical and public health purposes. Estimates of gestational age using fetal ultrasound measurements are considered most accurate but are frequently unavailable in low- and middle-income countries. The objective of this study was to assess the validity of last menstrual period and Farr neonatal examination estimates of gestational age, compared to ultrasound estimates, in a large cohort of women in Vietnam.

**Methods:**

Data for this analysis come from a randomized, placebo-controlled micronutrient supplementation trial in Vietnam. We analyzed 912 women with ultrasound and prospectively-collected last menstrual period estimates of gestational age and 685 women with ultrasound and Farr estimates of gestational age. We used the Wilcoxon signed rank sum test to assess differences in gestational age estimated by last menstrual period or Farr examination compared to ultrasound and computed the intraclass correlation coefficient (ICC) and concordance correlation coefficient (CCC) to quantify agreement between methods. We computed the Kappa coefficient (κ) to quantify agreement in preterm, term and post-term classification.

**Results:**

The median gestational age estimated by ultrasound was 273.9 days. Gestational age was slightly overestimated by last menstrual period (median 276.0 days, *P* < 0.001) and more greatly overestimated by Farr examination (median 286.7 days, *P* < 0.001). Gestational age estimates by last menstrual period and ultrasound were moderately correlated (ICC = 0.78) and concordant (CCC = 0.63), whereas gestational age estimates by Farr examination and ultrasound were weakly correlated (ICC = 0.26) and concordant (CCC = 0.05). Last menstrual period and ultrasound estimates of gestational age were within ± 14 days for 88.4% of women; Farr and ultrasound estimates were within ± 14 days for 55.8% of women. Last menstrual period and ultrasound estimates of gestational age had higher agreement in term classification (κ = 0.41) than Farr and ultrasound (κ = 0.05).

**Conclusion:**

In this study of women in Vietnam, we found last menstrual period provided a more accurate estimate of gestational age than the Farr examination when compared to ultrasound. These findings provide useful information about the utility and accuracy of different methods to estimate gestational age and suggest last menstrual period may be preferred over Farr examination in settings where ultrasound is unavailable.

**Trial registration:**

The trial was registered at ClinicalTrials.Gov as NCT01665378 on August 13, 2012.

**Electronic supplementary material:**

The online version of this article (doi:10.1186/s12884-016-1192-5) contains supplementary material, which is available to authorized users.

## Background

Accurate estimates of gestational age (GA) are important for both clinical practice and public health activities. Clinically, estimates of GA identify infants at risk for adverse health outcomes because GA is a proxy for fetal development and is associated with infant survival [1]. Public health indicators, such as proportion of preterm birth, rely on accurate estimates of GA to monitor population health, identify subgroups requiring intervention and evaluate public health programs [2].

Conceptually, GA refers to the duration of time between conception and delivery; because the timing of conception cannot be easily ascertained, GA is commonly estimated as the difference between the first day of the last menstrual period (LMP) and the delivery date [2]. Last menstrual period estimates of GA assume that the menstrual cycle occurs regularly and lasts 28 days, and that ovulation occurs on the 14^th^ day with conception occurring shortly thereafter; however, these assumptions may not apply to all women. Estimates of GA based on LMP are widely used because this information is easy and inexpensive to collect, but women may be unable to recall their LMP or may misreport their LMP due to mid-cycle bleeding or occasional bleeding during pregnancy [3]. Furthermore, the accuracy of LMP may decrease as recall length increases [4]. Women who are younger, primiparous, or have lower education are more likely to misreport LMP [3, 5] and in low- and middle-income settings, where educational attainment tends to be lower [6], it is possible that recall errors seriously influence the accuracy of reported LMP.

In settings where LMP may be biased, neonatal examinations may be used to estimate GA [3]. Neonatal examinations assess physical and/or neuromuscular maturity of newborn infants using standardized scoring methods and convert scores to estimates of GA; unlike LMP estimates of GA, neonatal examinations do not directly measure pregnancy duration. Neonatal examinations are typically used in clinical settings and rely on well-trained health care professionals to examine infants. Gestational age estimates from neonatal examinations have been found to be less accurate and reliable than other methods and estimates may vary by race/ethnicity [3, 7]. Furthermore, examinations that assess both physical and neuromuscular characteristics may be complex for clinicians and stressful for newborns, which may limit their utility [8].

Recently, it has become common to estimate GA using ultrasound measurements of fetal biometry; this is done by relating biometry measurements to GA through regression equations. First trimester measurements of crown-rump length provide the most accurate estimates of GA, with an estimated error of ± 5–7 days [9]. Accuracy of ultrasound estimates of GA decreases in the second trimester, with estimated error of ±10–14 days due to increased variability in fetal biometry [9]. Ultrasound estimates of GA are limited because actual pregnancy duration is not measured and this method assumes all variation in fetal size is attributable to GA, which does not account for normal variability [3]. Despite these limitations, first or second trimester ultrasound estimates of GA have been found to be more accurate when predicting delivery date compared to LMP-based estimates [3, 9–11].

In low- and middle-income settings, ultrasound estimates of GA are frequently not feasible due to limited resources or delayed entry into prenatal care [12]; thus, it is necessary to evaluate less expensive and more practical methods to estimate GA. To date, few studies have assessed estimates of GA based on LMP and neonatal examination compared to ultrasound in low- and middle-income countries. A study in Bangladesh concluded LMP was a more reliable method than neonatal examinations but findings were limited to infants younger than 33 weeks gestation [13]. A study in Guatemala found similar results, but was limited by a small sample size [14]. The objective of this study was to assess the validity of LMP and neonatal examination estimates of GA compared to ultrasound estimates in a large cohort of women in Vietnam.

## Methods

Data for this analysis (Additional file 1) come from the PRECONCEPT trial, a double-blind, randomized trial investigating the effects of pre-conceptual micronutrient supplementation on maternal and child outcomes in the Thai Nguyen province of Vietnam [15]. The PRECONCEPT trial is a collaboration between Emory University in the USA and the Thai Nguyen University of Medicine and Pharmacy in Vietnam and was approved by the Ethical Committee of Institute of Social and Medicine Studies in Vietnam and Emory University’s Institutional Review Board.

Women of reproductive age were enrolled into the PRECONCEPT trial if they were currently married, intended to remain in the study area, planned to have a child within one year but were not currently pregnant, did not regularly consume micronutrient supplements or did not have a history of high-risk pregnancy [15]. At enrollment, participants provided informed consent, and baseline demographic and anthropometric data were collected. Specifically, height was measured using a portable stadiometer and weight was measured using an electronic Seca scale; measurements were completed in duplicate and followed standard procedures [16, 17]. At enrollment, women were randomized into treatment groups and received biweekly supplements by village health workers who also monitored pregnancy status. Pregnancy was confirmed at local Commune Health Centers and women who conceived during the study period from 2012 to 2014 received prenatal care through the existing health system and were followed up for pregnancy outcomes. Information at delivery, including infant birth weight and length, were collected by study nurses or physicians. Infant weight was measured within 7 days of delivery using a UNICEF beam-type scale. Recumbent length at birth was measured using a wooden measurement board. Measurements were completed in duplicate [16, 17].

We estimated GA at delivery using three methods: LMP, the Farr neonatal examination (referred to as ‘Farr’) and ultrasound. The first day of the LMP was obtained prospectively by village health workers during biweekly home visits to distribute supplements and monitor pregnancy status. If LMP was reported five or more weeks prior to the visit, pregnancy status was confirmed and information on LMP was transferred to clinic staff to estimate GA. LMP estimates of GA at delivery were calculated by subtracting a woman’s LMP from her delivery date.

The Farr examination was used to estimate GA in the PRECONCEPT trial because it assesses only physical characteristics and is more practical than neonatal examinations that include neuromuscular assessments. The Farr examination scores 12 characteristics of physical maturity: skin texture, skin color, skin opacity, edema, lanugo, skull hardness, ear form, ear firmness, genitalia, breast size, nipple formation and plantar skin creases [18]. Each characteristic is scored from 0 to 4, with a higher score indicating advanced maturity; scores are summed and converted to weeks of completed gestation by equations developed by Farr and colleagues [19]. Study doctors or nurses completed Farr examinations at district hospitals within 24 h of birth.

We considered ultrasound estimates of GA as the gold standard method because previous validation studies have consistently found ultrasound estimates of GA to be more accurate than other methods [3, 9–11]. We calculated ultrasound estimates of GA in several steps. First, fetal femur length and head circumference were measured in replicate by trained study doctors using a portable ultrasound machine (Prosound 2, Hitachi Aloka Japan); if duplicate measurements differed by more than 4 mm for femur length (FL) or 10 mm for head circumference (HC), a third measurement was taken and the average of the two closest measurements were used for analysis. Next, GA on the day of the measurements was calculated using a regression equation recently published by Papageorghiou and colleagues [20]. Briefly, this equation was developed using second trimester fetal biometry measurements from a single ultrasound examination among women with certain LMP dates in the INTERGROWTH-21^st^ project. A machine learning approach was used to generate candidate equations relating combinations of fetal biometry variables (including head circumference, femur length, occipitofrontal diameter, abdominal circumference, and biparietal diameter measurements) to GA. A final equation was selected based upon minimization of prediction error, goodness of fit and model complexity. The final model that provided the best estimates of GA included fetal head circumference and femur length measurements; inclusion of additional fetal biometric measures did not improve prediction of GA. The regression equation relating head circumference and femur length to GA is as follows:

$$ \mathrm{l}\mathrm{o}{\mathrm{g}}_{\mathrm{e}}\left(\mathrm{G}\mathrm{A}\right)=0.03243 \times {\left[\mathrm{l}\mathrm{o}{\mathrm{g}}_{\mathrm{e}}\left(\mathrm{H}\mathrm{C}\right)\right]}^2 + 0.001644 \times \mathrm{F}\mathrm{L} \times \mathrm{l}\mathrm{o}{\mathrm{g}}_{\mathrm{e}}\left(\mathrm{H}\mathrm{C}\right) + 3.813 $$

Gestational age at delivery was estimated by adding the difference between the delivery date and the date of ultrasound measurement to the estimated GA on the day of ultrasound.

**Fig. 1:**
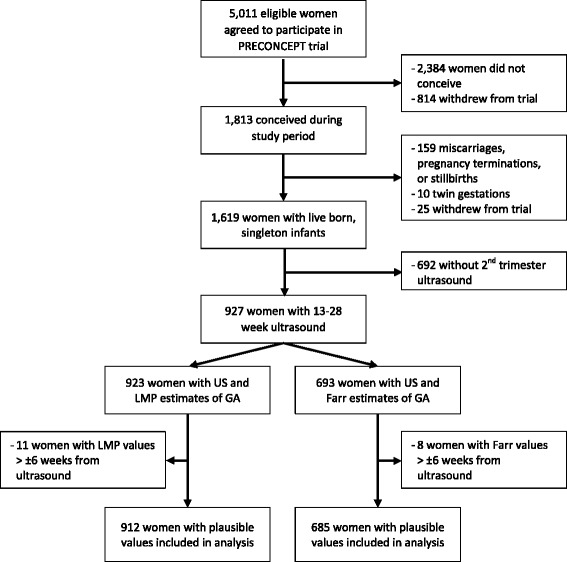
Flow chart describing sample of eligible women and women included in analysis from PRECONCEPT trial

Overall, 5011 women were eligible and agreed to participate in the PRECONCEPT trial (Fig. 1); of those, 2384 women did not conceive during the trial period and 814 withdrew from the trial before conceiving, resulting in 1813 pregnancies. A total of 1619 women gave birth to a live born, singleton infant, and we restricted our sample to 927 women with available second trimester (13–28 week) ultrasound (Fig. 1). To maximize sample size, two subsamples were created: i) 923 women with second trimester ultrasound and LMP estimates of GA and ii) 693 women with second trimester ultrasound and Farr estimates of GA. Women with LMP (*n* = 11) or Farr estimates (*n* = 8) of GA that differed from ultrasound estimates by more than six weeks were considered outliers and excluded from analysis. Our final subsample sizes were 912 women for the LMP analysis and 685 women for the Farr analysis.

We examined the validity of LMP and Farr examination estimates of GA compared to ultrasound estimates (considered gold standard) in several ways. We used the median and interquartile range to describe the distribution of GA estimated by each method and the Wilcoxon signed rank sum test to assess statistically significant differences between methods because GA distributions estimated by LMP, Farr and ultrasound were slightly skewed. We also quantified the difference between methods using the mean difference (LMP – ultrasound or Farr – ultrasound) because the distribution of the differences followed an approximately normal distribution. The intra-class correlation coefficient (ICC) was used to estimate consistency between methods and was computed using a two-way, random effects analysis of variance model; a higher ICC indicates a higher degree of consistency [21, 22]. We used the concordance correlation coefficient (CCC) to quantify the absolute agreement between two methods; it is visualized as the degree in which measurements from two methods fall on a line that intersects the origin at 45° (the line of perfect concordance). A CCC of 1 indicates perfect concordance [23]. Finally, we used the Kappa coefficient to examine the agreement adjusted for chance in classification of preterm (<259 days), term (259–294 days) and post-term (>294 days) births between LMP or Farr estimates of GA compared to ultrasound.

To visually examine our data, we plotted the distributions of GA estimated by LMP or Farr compared to ultrasound using Bland-Altman plots. The difference between GA estimation methods (LMP – ultrasound or Farr – ultrasound) varied across average GA; therefore, we used a regression approach to determine the mean difference as a function of average GA and to determine the 95% Limits of Agreement; the Limits of Agreement may be interpreted as the range where 95% of differences are expected to occur [24].

We conducted a sensitivity analysis to examine the validity of LMP and Farr examination estimates of GA, compared to ultrasound estimates, separately for males and females. Statistical analyses were conducted using SAS version 9.3 (SAS Institute, Cary, NC). *P* < 0.05 was considered statistically significant.

## Results

Maternal and infant characteristics for 912 women with ultrasound and LMP estimates of GA are shown in Table 1; characteristics were similar for 685 women with ultrasound and Farr estimates of GA (data not shown). On average, women were 28 years old and 30.8% had a body mass index (BMI; kg/m^2^) below 18.5. One-half of infants were male and, on average, infants were 49 cm long at birth and 5% were classified as low-birth-weight (<2500 g). We found no significant differences in maternal or infant characteristics between women included and excluded in the analysis (data not shown).

**Table 1:** Maternal and infant characteristics of the study sample (*n* = 912)

Characteristic	Mean (SD) or n (%)
Maternal age (years)	27.6 (4.3)
Maternal pre-pregnant weight (kg)	45.7 (5.3)
Maternal height (cm)	152.7 (5.0)
Maternal pre-pregnancy BMI (kg/m^2^)	19.6 (2.0)
Maternal underweight (<18.5 kg/m^2^)	280 (30.8)
Number of children^a^	
0	32 (4.0)
1	752 (94.6)
≥ 2	11 (1.4)
Sex of infant (Male)	457 (50.1)
Birthweight (g)	3098.3 (457.5)
Birth length (cm)	49.0 (3.1)
Low birthweight (<2500 g)	45 (5.0)
High birthweight (>3500 g)	123 (13.6)

^a^Total may not add to 912 due to individuals with missing information

**Fig. 2:**
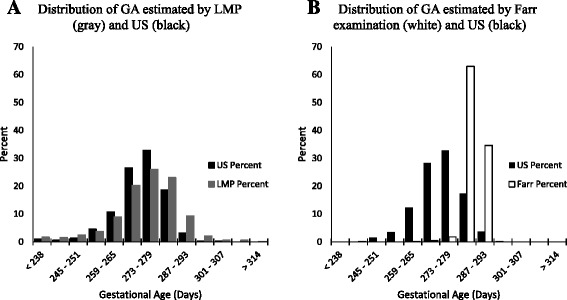
Distribution of gestational age (GA) estimated by last menstrual period (LMP), Farr examination and ultrasound measurements (US). **a** Distribution of GA estimated by LMP (*gray*) and US (*black*). **b** Distribution of GA estimated by Farr examination (*white*) and US (*black*)

**Fig. 3:**
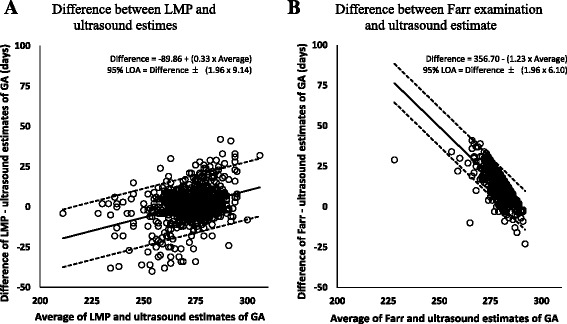
Bland-Altman plots depicting difference between (**a**) last menstrual period (LMP) and ultrasound estimates of gestational age or (**b**) Farr examination and ultrasound estimates of gestational age, plotted against the average of the two methods. Solid line indicates mean difference and dotted line indicates Bland-Altman 95% Limits of Agreement (LOA). Positive y-values indicate overestimation by (**a**) LMP estimates or (**b**) Farr estimates compared to ultrasound

The GA distributions estimated by LMP and Farr compared to ultrasound are displayed in Fig. 2a and b, respectively. Generally, the GA distributions estimated by LMP and ultrasound appeared similar and overlap, although LMP slightly overestimate GA. The GA distribution estimated by Farr appeared narrower than that estimated by ultrasound and also appeared to overestimate GA. These trends are confirmed in the Bland-Altman plots for LMP compared to ultrasound and Farr compared to ultrasound in Fig. 3a and b, respectively. Specifically, compared to ultrasound, LMP underestimated GA for infants born earlier and overestimated GA for infants born later (Fig. 3a); the Farr method frequently overestimated GA, especially for infants born earlier (Fig. 3b).

The agreement between GA estimation methods is presented in Table 2. The median GA estimated by ultrasound was approximately 274 days. The median GA estimated by LMP and Farr were approximately 276 and 287 days, respectively, and were significantly greater than GA estimated by ultrasound (*p* < 0.001). On average, compared to ultrasound, LMP overestimated GA by 1.4 days (95% CI: 0.7–2.0) whereas Farr overestimated by 12.9 days (95% CI: 12.2–13.5). The LMP and ultrasound estimates of GA were moderately correlated (ICC = 0.78, 95% CI: 0.74–0.80) and moderately concordant (CCC = 0.63, 95%CI: 0.59–0.67). In contrast, the Farr estimate was weakly correlated with ultrasound estimate (ICC = 0.26, 95% CI: 0.15–0.37) and showed low concordance (CCC = 0.05, 95% CI: 0.04–0.07). Last menstrual period and ultrasound estimates of GA were within ± 7 days for nearly 69% of women and within ± 14 days for approximately 88% of women (Table 2). Farr and ultrasound estimates of GA were within ± 7 and ±14 days for approximately 24% and 56% of women, respectively (Table 2).

**Table 2:** Agreement in gestational age estimated by last menstrual period (LMP) and Farr examination compared to ultrasound

Measure	Ultrasound	LMP (*n* = 912)	Farr examination (*n* = 685)
Median GA (days) (Interquartile range)	273.9 (268.2, 279.3)	276.0^a^ (268.0, 282.0)	286.7^a^ (286.7, 288.6)
Mean difference (days) (95% CI)	Reference	1.4 (0.7, 2.0)	12.9 (12.2, 13.5)
Intra-class correlation coefficient (95% CI)	Reference	0.78 (0.74, 0.80)	0.26 (0.15, 0.37)
Concordance correlation coefficient (95% CI)	Reference	0.63 (0.59, 0.67)	0.05 (0.04, 0.07)
Agreement with ultrasound n (%)			
±7 days	Reference	625 (68.5)	166 (24.2)
±10 days	Reference	731 (80.2)	254 (37.1)
±14 days	Reference	806 (88.4)	382 (55.8)

^a^ *P* < 0.001

Table 3 depicts the agreement in the classification of preterm, term and post-term infants estimated by LMP or Farr methods compared to ultrasound. Compared with ultrasound estimates of GA, LMP classified slightly more infants as preterm (9.3% vs 7.5%) and post-term (2.6% vs 0.5%); Farr estimates classified almost all infants as term (99.9%) and did not classify any infants as post-term. Overall, LMP and ultrasound had higher agreement in term classification (κ = 0.41, *p* < 0.001) than Farr and ultrasound (κ = 0.05, *p* < 0.001).

**Table 3:** Agreement in preterm, term and post-term classification by last menstrual period (LMP) or Farr examination estimates of gestational age compared to ultrasound estimates

	Ultrasound classification				Agreement statistics	
LMP classification	Preterm n (%)	Term n (%)	Post-term n (%)	Total n (%)	Crude % agreement	Kappa statistic
Preterm	41 (4.5)	44 (4.8)	0 (0.0)	85 (9.3)	89.0	0.41^a^
Term	27 (3.0)	770 (84.4)	4 (0.4)	801 (87.8)		
Post-term	0 (0.0)	25 (2.7)	1 (0.1)	26 (2.6)		
Total	68 (7.5)	839 (92.0)	5 (0.5)	912		
Farr classification						
Preterm	1 (0.2)	0 (0.0)	0 (0.0)	1 (0.2)	94.5	0.05^a^
Term	35 (5.1)	646 (94.3)	3 (0.4)	684 (99.9)		
Post-term	0 (0.0)	0 (0.0)	0 (0.0)	0 (0.0)		
Total	36 (5.3)	646 (92.3)	3 (0.4)	685		

^a^ *P* < 0.001

In sensitivity analyses examining estimates of GA for males and females separately, we found no substantial differences in estimates by gender, nor did estimates substantially differ from aggregate results (data not shown).

## Discussion

This was one of the few studies that examined the validity of LMP and neonatal examination estimates of GA compared to ultrasound in a low- and middle-income country. Overall, we found LMP provided a better estimate of GA than the Farr examination. Compared to ultrasound, LMP overestimated mean GA by 1.4 days while the Farr examination overestimated mean GA by 12.9 days. In addition, over 88% of women had LMP and ultrasound estimates of GA within ± 14 days compared to 56% with Farr and ultrasound estimates within ± 14 days. LMP and ultrasound estimates of GA also had higher correlation, concordance and agreement in term status than did Farr compared to ultrasound estimates of GA.

Our finding that LMP more accurately estimated GA than neonatal examinations is consistent with findings from a study in Guatemala, which found no significant difference in mean GA estimated by ultrasound or LMP and found 94% of women had ultrasound and LMP estimates of GA within ± 14 days. Neonatal examination in the Guatemala study underestimated mean GA by over 3 days and 82% of women had neonatal examination and ultrasound estimates of GA within ± 14 days [14]. Notably, LMP was ascertained prospectively in both the Guatemala study and in our study, which likely minimized recall bias and improved the performance of LMP estimates of GA.

Results from the Guatemala study suggest LMP and neonatal examination were more valid in that setting than was observed in our study; this may be due to the greater extent of intrauterine growth restriction (IUGR) in our study population and different neonatal examinations used in each study. Findings from a recent study by our group indicate a pattern of IUGR among women participating in the PRECONCEPT trial, which began in mid-pregnancy and continued through delivery [25]. Ultrasound measurements completed earlier in pregnancy are also less likely influenced by IUGR [10]; in our study, measurements were completed before 28 weeks while in the Guatemala study, all measurements were completed before 24 weeks. Taken together, ultrasound estimates of GA in our study were more likely to be influenced by IUGR, which would underestimate GA and may bias our results. Moreover, estimates of GA by neonatal examination may vary by race/ethnicity and may explain differences in validity observed in our study and the Guatemala study [7]. Studies in other low- and middle income countries have also found the Farr examination to be less accurate than other neonatal examinations [26].

In addition to improved accuracy over neonatal examinations, LMP estimates of GA may be preferred because healthcare professionals can prioritize care for mothers and newborns rather than conduct the neonatal examination. Indeed, one study in Bangladesh found LMP estimates of GA were slightly less correlated and concordant with ultrasound than two neonatal examination methods; nevertheless, the authors conclude LMP was valid and clinically preferred to estimate GA in a low-resource setting [13]. Measurement of the symphysis-fundal height is an alternate method that may be used to estimate GA during pregnancy, but there is inconsistent evidence whether symphysis-fundal height performs better than LMP in low- and middle income countries [27].

Our study is strengthened by a large sample size, which allowed us to detect a mean difference of less than 2 days between LMP and ultrasound estimates of GA and allowed us to conclude that that LMP is a reasonable alternative to US estimates of GA in our study and possibly other low- and middle-income settings. Ultrasound estimates of GA were calculated from equations recently developed using a machine learning algorithm to identify the best set of fetal biometric predictors of GA [20]. Further, dating equations were derived using data from the INTERGROWTH-21st Project, which utilized a large, multi-site, population-based design with strict quality control measures to ensure internal validity [28]. Finally, our measure of LMP was assessed prospectively and likely reduced recall errors; however, this may not represent usual circumstances in other low- and middle-income countries. Our study also has some limitations. Specifically, we are limited by the timing of fetal ultrasound measurements. First trimester ultrasound measurements are optimal when estimating GA because of limited variability in fetal size due to IUGR [3]; ultrasound estimates in our study are likely influenced by IUGR, which would underestimate GA and may bias results. In low- and middle-income countries, however, first trimester ultrasound measurements are typically not feasible and previous studies have demonstrated the accuracy of second trimester ultrasound estimates of GA [29]. Despite being considered gold standard, it is important to recognize that ultrasound estimates of GA are not direct measurements of pregnancy duration and, similar to other GA estimation methods, are subject to some error [3]; importantly, studies have established the improved accuracy of ultrasound estimates of GA compared with other methods [3, 9–11].

## Conclusion

LMP estimates of GA performed better than Farr examination when compared to ultrasound in a population of Southeast Asian women. As ultrasound measurements are frequently not available in low- and middle-income countries, it is important to identify alternative methods that provide accurate estimates of GA. Our findings provide information regarding the utility of LMP-based estimates of GA compared to Farr examination estimates, and the level of accuracy compared to ultrasound estimates.

## Additional file

Additional file 1: Analytic Dataset. (XLSX 123 kb)

## Abbreviations

BMI: Body mass index
CCC: Concordance correlation coefficient
Farr: Farr neonatal examination
FL: Femur length
GA: Gestational age
HC: Head circumference
ICC: Intra-class correlation coefficient
IUGR: Intrauterine growth restriction
LMP: Last menstrual period
